# Applications of Biodegradable Magnesium-Based Materials in Reconstructive Oral and Maxillofacial Surgery: A Review

**DOI:** 10.3390/molecules27175529

**Published:** 2022-08-28

**Authors:** Sanja Vujović, Jana Desnica, Dragana Stanišić, Irena Ognjanović, Momir Stevanovic, Gvozden Rosic

**Affiliations:** 1 Department of Dentistry, Faculty of Medical Sciences, University of Kragujevac, Svetozara Markovića 69, 34000 Kragujevac, Serbia; 2 Department of Physiology, Faculty of Medical Sciences, University of Kragujevac, Svetozara Markovića 69, 34000 Kragujevac, Serbia

**Keywords:** magnesium, biodegradable metals, maxillofacial surgery, guided bone regeneration, bone fracture

## Abstract

Reconstruction of defects in the maxillofacial region following traumatic injuries, craniofacial deformities, defects from tumor removal, or infections in the maxillofacial area represents a major challenge for surgeons. Various materials have been studied for the reconstruction of defects in the maxillofacial area. Biodegradable metals have been widely researched due to their excellent biological properties. Magnesium (Mg) and Mg-based materials have been extensively studied for tissue regeneration procedures due to biodegradability, mechanical characteristics, osteogenic capacity, biocompatibility, and antibacterial properties. The aim of this review was to analyze and discuss the applications of Mg and Mg-based materials in reconstructive oral and maxillofacial surgery in the fields of guided bone regeneration, dental implantology, fixation of facial bone fractures and soft tissue regeneration.

## 1. Introduction

Reconstruction of defects in the maxillofacial region following traumatic injuries, craniofacial deformities, defects from tumor removal or infections in the maxillofacial area represents a major challenge for surgeons. The maxillofacial region has a significant impact on patients’ well-being, and any facial deformity or dysfunction has a devastating effect on the patients’ quality of life [1,2]. Reconstruction or augmentation of craniofacial bones is one of the most frequent surgical procedures in maxillofacial surgery. After blood transfusion, bone grafting is the second-most common tissue transplantation procedure worldwide [3]. Extensive clinical research on bone grafting and augmentation with autografts, allografts and xenografts has been performed. Autografts taken from the same patient are considered the gold standard for bone reconstruction, since no immune reaction is expected. However, the need for additional surgical intervention, donor site morbidity, limited bone availability and significant graft resorption emphasized the need for different bone substituents [4,5]. Allografts taken from genetically non-identical members of the same species carry the risk of pathogen transfer and immune system rejection [6]. Xenografts, usually bovine-derived, are most often used to augment intraoral bone defects [7]. However, the application of animal-derived materials to humans has certain limitations concerning patients’ religion, dietary restrictions and ethical controversy [7]. To overcome these drawbacks, extensive research on bone tissue engineering using bio-mimicking, resorbable and biocompatible bone substitutes has been performed in the past years. These synthetic bone substitutes serve as an artificial extracellular matrix to promote bone healing until they are partially or completely replaced by newly formed bone [8,9]. Biodegradable polymers are extensively studied as bone scaffolds and have proven osteoconductive properties as well as excellent biocompatibility [8,9]. However, low mechanical strength, unstable rates of degradability and immune reaction to products of polymer degradation limit their use in clinical practice [10]. Titanium (Ti) is the most commonly used non-biodegradable metal in maxillofacial surgery for stabilization of fractures or osteotomies, dental implantation procedures and guided bone regeneration (GBR). However, Ti-based materials are bioinert, and secondary surgical intervention is often needed to remove the Ti materials from the organism mainly due to discomfort or surgical site infection, which may occur in up to 33% of cases [11].

Biodegradable metals have been extensively studied for tissue regeneration procedures due to their biodegradability, mechanical properties, osteogenic capacity, biocompatibility and antibacterial properties [12]. Magnesium (Mg)-based materials have been used in medicine since the 19th century. Mg is an essential metal for the human organism, and it is involved in more than 300 cell enzymatic reactions, mitochondrial activity, protein translation, DNA synthesis and cell proliferation [13]. About 60% of Mg in a healthy adult is deposited in bones [13]. Mg is resorbed from the intestines, and its homeostasis in the organism depends on renal function [13]. Due to its mechanical properties, an elastic modulus similar to human bones and its biosafety, Mg had been used in orthopedic surgery in the early 20th century until it was replaced with Ti materials [14,15]. The elastic modulus of Mg is about 45 GPa which is much closer to that of cortical bone (10–23 GPa) compared to Ti [16]. The development of bioresorbable Mg-based materials prevents the need for second-stage surgery for the removal of implanted material and associated comorbidities.

The aim of this review is to analyze and discuss the applications of Mg and Mg-based materials in reconstructive oral and maxillofacial surgery. The review is divided into subheadings about the use of Mg-based material in: (a) GBR, (b) dental implant coatings, (c) immobilization of facial bone fractures, and d) soft tissue regeneration. Based on the literary data, we will discuss possibilities and directions for future development and applications of Mg-based materials in oral and maxillofacial surgery.

## 2. Biological Properties of Mg-Based Materials

The biodegradability of magnesium-based materials is the major advantage of Mg materials. Mg corrodes in the physiological environment and releases species such as Mg ions (Mg^2+^), alloying elements, H_2_ gas, and OH^−^ [9]. In an alkaline environment, magnesium hydroxide Mg(OH)_2_ is deposited on the Mg matrix and forms a protective layer [10]. In the case of fast degradation and corrosion of Mg-based materials, a locally high concentration of Mg ions disturbs calcium-mediated bone reparation and regeneration processes. Products of corrosion of Mg-based materials such as magnesium hydroxide and hydrogen gas may impair tissue healing due to the formation of gas cavities and compression to surrounding soft tissues [17,18].

Due to the roles of Mg in major cellular functions, magnesium-based materials in the forms of bone cement, bone scaffolds, and implant coatings were evaluated as promising candidates for bone regeneration therapies. Various in vitro studies reported Mg ions to have positive effects on bone cells, including enhanced proliferation, migration and alkaline phosphatase activity, increased differentiation capacity of human osteoblast cells, and increased proliferation of bone marrow-derived stromal cells (BMSCs) [17,18,19]. Having in mind that Mg-based materials are biodegradable, the osteogenic effect of Mg ions is dose-dependent. Concentrations of Mg ions in tissue ranging from 2.5–10 mM have a positive effect on the proliferation and differentiation of human BMSCs [19,20]. However, higher concentrations of Mg ions in the tissue were connected with decreased mineralization capacity and matrix deposition of BMSCs [21,22]. The inhibitory effect on osteogenesis of a high local Mg concentration in tissue was linked with alteration in calcium metabolism in cells due to competition between calcium and magnesium ions for the same ion transporters and the inhibition of expression of the calcium-sensing receptor [22,23]. This resulted in a decreased intracellular calcium concentration and decreased calcium influx in cells [22,23].

Investigations on the implementation of Mg-based materials found no adverse effects on health. The resorption of Mg results in elevated local concentrations of ions, which is rarely harmful to cells because cells can handle concentrations of Mg about 16-times higher than the physiological ones [10]. Upon implantation in the organism, degradation of Mg does not result in increased Mg deposition in lymph nodes [24]. In vivo studies reported that there were no health risks following Mg implantation in rats with chronic renal failure [25]. The results of an in vivo study indicated that Mg absorption, after implantation of Mg alloy rods, at the degradation rate of 2.32 mm/yr did not lead to dysfunction of the heart, liver, kidney, and spleen of the rabbits [26]. Moreover, Mg alloy rods inserted in the femoral bone of the New Zealand rabbits did not cause changes in the Mg serum levels, kidney and liver function, and histological structure of the vital organs, like the heart and spleen [27]. Clinical trials following the implantation of Mg screws for the treatment of orthopedic fractures found no signs of hypermagnesemia and demonstrated normal levels of Mg blood concentration [3]. Also, no complications, such as allergic reactions, liver/renal dysfunction, or an increase in Mg serum levels, were observed after the application of Mg alloy compressive screws in patients undergoing corrective orthopedic surgeries [28].

## 3. Bioresorbable Mg-Based Materials for Guided Bone Regeneration (GBR)

### 3.1. GBR Membranes

GBR in the maxillofacial region has been extensively studied over the past decades. Loss of jaw bones due to periodontitis, tooth extractions, operation on tumors and cysts, systemic diseases or infections results in different jaw abnormalities and changes to the occlusion. GBR comprises the use of bone scaffolds or substituents and biomembranes in order to augment bone defects and induce osteogenesis [29].

**Figure 1:**
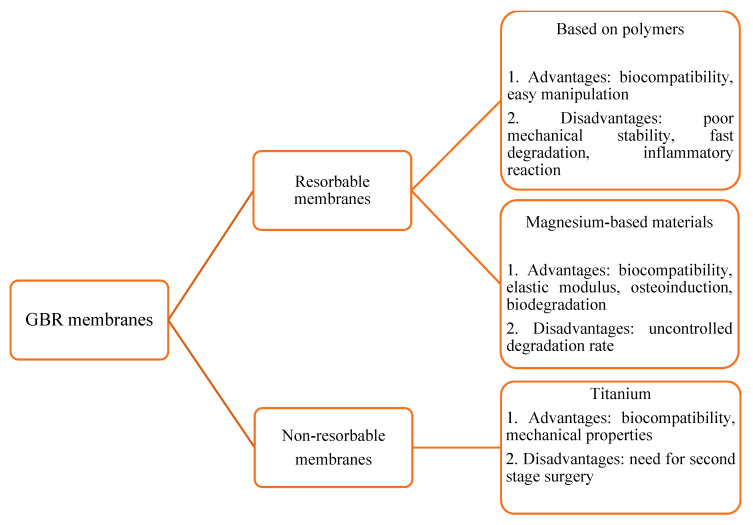
Guided bone regeneration membranes (GBR) for bone tissue regeneration.

Biomembranes act as a barrier between hard and soft tissues. They prevent the soft tissues from interfering with osteogenesis, thus providing enough space for the differentiation of osteoprogenitor cells. Biomembranes used in clinical practice could be resorbable based on synthetic (poly(lactic-*co*-glycolic acid) (PLGA), polyethyleneimine (PEI), poly(L-lactic acid) (PLLA)) or natural (collagen, chitosan) polymers, non-resorbable (Ti mesh, or polytetrafluoroethylene (e-PTFE)) (Figure 1).

Resorbable membranes are widely used due to their economic benefits, biocompatibility and easy manipulation. However, these membranes are often deformed due to rapid degradation, which may impair bone regeneration, while their low mechanical strength makes them unsuitable for larger bone defects [29]. On the other hand, the application of non-resorbable membranes implies the need for second-stage surgery. Biomembranes with Mg-based materials could combine the mechanical strength of metallic alloys, biocompatibility and slow degradation in natural tissues as a promising solution for this problem. Furthermore, the mechanical properties of Mg alloys allow the membrane to maintain the space for osteogenesis and bone height in alveolar sockets or large bone defects [30]. The good plasticity of Mg alloys is useful in handling and adapting membranes to complex shapes of bony defects [31]. In addition, the antibacterial properties of Mg alloys reduce the risk of bacterial infection and bone resorption [32].

Reports on the clinical application of Mg-based GBR membranes are scarce due to difficulties in adapting their degradation rate to clinical expectations. A recent in vivo study evaluated the Mg-alloy GBR membrane (Mg-2Zn-0.46Y-0.5Nd) for bone healing in a critical-sized mandibular bone defect within a study with beagle dogs [33]. The results of this research showed good biocompatibility, osteoconduction and osteogenic potential of the membrane. However, the authors observed almost complete postoperative resorption of the membrane within 3 months, which led to reduced osteogenic effect in later phases. Similarly, the results of the in vivo study with a mineralized collagen/Mg–Ca alloy combined scaffold designed to withstand the physiological forces in the mouth did not achieve the desired restoration of alveolar bone defects in dogs [34].

In order to decrease the process of degradation, various coatings to Mg materials were added. In an in vitro–in vivo study on the critical-sized defect of rabbit calvaria, the Mg-Zn-Gd membrane coated with calcium-phosphate (Ca-P) showed superior osteogenic and mechanical properties compared to the non-coated Mg-Zn-Gd membrane [35]. Surface modification using plasma electrolytic oxidation and hydrothermal treatment on Mg mesh resulted in decreased degradation and better quality of newly formed bone in calvaria defects in rats during an in vivo experiment [36]. Complex hybrid membrane AZ31-PLGA-demineralized bone matrix (DBM) had a strong ability to promote the proliferation of bone marrow stem cells and resulted in excellent repair of the critical-sized calvaria defect model, reported by in vivo research [37]. A similar in vitro–in vivo study showed that the addition of pure Mg particles to the PLGA scaffold in order to overcome the low mechanical strength of PLGA resulted in significant proliferation of BMSCs and significantly increased bone formation in a canine premolar tooth socket [38].

Composite Mg-polymer membranes and materials showed promising results in bone repair. Photo-cross-linkable collagen/polycaprolactone methacryloyl/magnesium (Col/PCLMA/Mg) composite membranes demonstrated excellent mechanical properties and elastic modulus, biocompatibility, and promoted cell attachment and osteoprogenitor cell proliferation when implanted into calvaria bone defects of rats for 8 weeks in an in vitro–in vivo study [39].

### 3.2. Mg-Based Scaffolds for GBR

Bone tissue is a natural composite mixture of organic (collagen fibers) and inorganic substances (hydroxyapatite crystals) [2]. Composite scaffolds combining the advantages of biodegradable polymers such as PLGA and PEI with hydroxyapatite (HA) ceramics have been extensively studied because they resemble the natural bone structure, and its mechanical and osteoconductive properties are enhanced by a thin biodegradable polymer coating [4]. In vivo studies with composite HA–polymer scaffolds resulted in complete repair of a critical-sized defect in rabbit’s calvaria, a large defect of rabbit’s ulna, as well a critical size mandibular defect in swine [2,4,40]. However, due to the insufficient mechanical properties of composite bone scaffolds, deformation and brittle fracture may occur [41]. For this reason, Mg-based materials with excellent biocompatibility and mechanical properties were incorporated into HA to enhance their biological and physicochemical properties. In vitro and in vivo experiments demonstrated significantly improved HA properties with the addition of Mg [42]. The addition of Mg to HA resulted in improved chemical properties compared to stoichiometric HA, such as reduced crystallinity, high specific surface area, and enhanced solubility in natural tissues. These factors lead to improved cell adhesion, proliferation, and metabolic activity [43]. A mixture of HA and β-TCP doped with Mg (magnesium-doped biphasic calcium phosphate) mimics the natural inorganic bone matrix with excellent physicochemical properties [44]. Furthermore, the presence of Mg ions during synthesis also improves the thermal stability of HA and produces a more stable phase composition after heat treatment, which enables the production of porous or granulated scaffolds for biomedical applications, including oral and maxillofacial surgery and orthopedics [44,45]. Magnesium Hydroxyapatite (MgHA) scaffold was analyzed for bone regeneration in alveolar critical-sized bone defects in several animal and human trials. The results suggest that the MgHA scaffold could be a very effective bone substitute [46]. Various in vitro studies reported excellent biocompatibility for several cell lines [47,48,49]. Sartori et al. demonstrated in an in vivo study conducted on sheep that MgHA provides osteoconductive structural support during the process of bone regeneration [50]. Santos et al. concluded in an in vivo experiment that MgHA, when implanted in a critical bone defect in rat calvaria, is a biocompatible and osteoconductive biomaterial [51]. A clinical study by Grigolato et al. showed that MgHA, used as a bone substitute in a mandibular defect due to ameloblastoma, exhibits excellent biological behavior and high osseointegration potential [52]. MgHA is a relatively well-studied Mg-based bone substitute material, and there are several commercial products researched for the reconstruction of maxillofacial bone defects.

Teeth extractions cause significant changes in the dimensions of the alveolar ridge due to resorption of the alveolar socket, which may impair dental implantation and prosthetic reconstruction [53]. Resorption of the alveolar socket is rapid following tooth extraction due to loss of function, and about 40–60% of bone is resorbed in the first two years [54]. The preservation of the alveolar socket volume following tooth extractions and alveolar ridge preservation or augmentation could be achieved using MgHA scaffolds. In a clinical study by Crespo et al. a split-mouth design was used to compare histologic and histomorphometric results of MgHA and porcine bone grafts for the preservation of fresh dental sockets [55]. The results of this study showed similar biologic behavior in bone formation and resorption processes. A similar clinical study that compared radiographic and histomorphometric results of MgHA and calcium sulfate grafts in fresh sockets after tooth extractions found a lower reduction of the alveolar ridge, more bone formation and more residual implant material in the MgHA group [56].

A prospective 2-year clinical study evaluated the survival of dental implants loaded 14 weeks after vertical alveolar ridge augmentation with nano-structured MgHA covered with Ti-polytetrafluoroethylene (e-PTFE) membrane [54]. The results of this study suggested that vertical ridge augmentation around Ti implants using MgHA can be successful in cases with early implant loading. However, an in vivo animal study with canines did not find a significant effect of MgHA on alveolar socket preservation and osseointegration of implants placed immediately into extraction sockets [57]. Recently, a clinical study investigated the effectiveness of a biomimetic MgHA/collagen-based bone substitute for alveolar socket preservation compared to deproteinized bovine bone matrix [58]. The results after 6 months showed similar vertical and horizontal alveolar ridge resorption, similar new bone formation between the groups and a significantly higher residual material for deproteinized bovine bone matrix. Crespo et al. compared the use of MgHA and autologous bone graft for maxillary sinus lift procedures [59]. The results of this clinical study suggested MgHA as a possible alternative to autologous bone graft for sinus lift operations.

There are promising results from using bovine bone grafts enriched with Mg for bone regeneration. An in vivo study on the biological properties of bovine xenogeneic biomaterial enriched with Mg on the healing of critical-sized defects on rat calvaria showed Mg biomaterial demonstrated osteoinductive properties and biodegradability during healing [60]. Similar results were reported for the rabbit calvaria defect repair in an in vivo study [61].

Mg-based bone types of cement have been used in orthopedics for bone and tendon repair [62]. The results of canine in vivo study that evaluated Mg-based bone cement for bone grafting of immediate implantation of extraction sockets showed success at filling in the bone defects without implant loss during the observation period [63]. However, the use of Mg-based bone types of cement may be doubtful due to their 3D structure and lack of porosity, which enables osteoconductive properties [10].

Zhang et al. developed 3D gel printing in an in vitro–in vivo study and used it to prepare an Mg scaffold with a controllable pore structure, and its surface was modified with a calcium phosphate coating [41]. The addition of calcium phosphate coating onto the surface of materials improved biocompatibility and biosafety, osteogenic induction and angiogenic ability; in addition, the degradation rate of materials can be effectively controlled by adjusting the thickness of calcium phosphate coating [64].

## 4. Mg and Mg-Based Materials for Ti Implant Coating

Surface characteristics of Ti implants have a major impact on the process of implant osseointegration, and research in the field of implant surface modification is important despite good and predictable rates of implant success [65]. Various surface coatings on dental implants were investigated in order to improve implant surface for stronger micromechanical retention and improved biological processes for osteogenesis [65,66]. Surface coating with bioceramics such as hydroxyapatite, calcium phosphate, and bioactive glass resulted in improved osseointegration. However, the practice has significant complications due to poor mechanical strength, brittleness and bacterial infections around implants [67].

Mg and Mg-based materials were studied as possible implant surface coatings due to elastic modulus of the material, osteogenic effect, biocompatibility and biodegradation of these materials. Results of in vitro and in vivo studies found positive effects of Mg coatings such as Mg carbonate, Mg fluoride, Mg oxide, Mg silicate, and HA incorporated with Mg and Zinc (Zn) [65]. The results of in vitro studies demonstrated that Ti implants coated with Mg-based coatings showed enhanced BMSCs proliferation and increased expression of osteogenic markers (alkaline phosphatase, osteocalcin, osteopontin, bone sialoprotein, RUNX-2), increased collagen type I deposition and antibacterial activity [68,69,70,71,72,73,74]. In an in vivo animal study comparing antibacterial properties of Mg and Mg-Zn co-implanted, Yu et al. showed both surfaces to have an excellent antibacterial effect against specific periodontal pathogens, such as *Porphyromonas gingivalis*, *Streptococcus mutans* and *Fusobacterium nucleatum* [68]. Additionally, another study found that MgO-HA and MgF_2_-HA coatings had a significantly better antibacterial effect against *Enterococcus* spp., *Micrococcus* spp. and *Candida albicans* than HA coatings [72].

New bone formation is quantitatively measured with the metrics of bone–implant contact and bone area. The results of in vivo studies revealed improved osseointegration, better new bone architecture, higher bone volume/total volume and bone-to-implant ratio with Mg coatings than conventional Ti surfaces [75,76,77,78]. Cho et al. found in a study on rabbits that the concentration of Mg ions had a significant effect on osseointegration since implants coated with 9.24% Mg had remarkably better removal torque value, bone–implant contact, bone fill area and new bone formation [75].

The results of in vitro studies clearly demonstrated Mg coatings had positive effects on osteoblastic differentiation of BMSCs, and increased cell proliferation and induction of osteogenesis to obtain implant osseointegration. In vivo studies showed that Mg coatings resulted in increased new bone formation, higher values of new bone and better new bone architecture [78]. Clinical studies are needed to confirm further clinical effects of Mg surface coatings.

## 5. Bioresorbable Mg-Based Materials for Osteosynthesis of the Facial Bone Fractures

Resorbable Mg-based materials have been extensively studied for use in orthopedic surgery since the beginning of the 20th century due to their biological properties and the elastic modulus similar to natural bone [79]. However, they were replaced by bioinert Ti materials due to superior mechanical properties for the treatment of complicated and load-bearing fractures. Since the implantation of Ti plates and screws for fracture immobilization requires a secondary surgical intervention and removal of Ti material due to infection, discomfort or plate exposure, bioresorbable Mg-based materials were de novo analyzed for the treatment of traumatic bone injuries [11]. Pre-clinical and clinical studies were mostly performed for the stabilization of orthopedic fractures, while limited data are available for the treatment of maxillofacial injuries.

For orthopedic injuries, both pure Mg and its alloys were evaluated. The biological and mechanical properties of Mg and its alloys are mainly influenced by material behavior in the tissue following implantation. In the natural conditions in the tissue, Mg corrodes and releases Mg ions and H_2_ gas into surrounding tissues and may cause significant emphysema in the rapid corrosion process [79]. There are pieces of evidence that H_2_ may induce osteogenesis and reduce osteoclastogenesis and thus benefit bone reparation [80]. The corrosion of Mg and Mg alloys depends on material structure (heterogeneity, metal purity and microstructure of the alloy), mechanical loads, pH of the surrounding tissues and vascularization [81,82,83]. The corrosion rate and degradation of Mg and its alloys are the main factors influencing their clinical application [81,82,83]. Pure Mg (99.99%) has a low corrosion rate and low mechanical strength [84]. However, pre-clinical studies found pure Mg promotes osteogenesis and fracture healing on rabbit femoral condyle fractures using Mg screws [84]. Mg alloys with rare earth elements (RE) such as scandium (Sc), yttrium (Y), gadolinium (Gd), zirconium (Zr) and neodymium (Nd) were synthesized in order to decrease corrosion and reduce degradation rate of 99.99% pure Mg. The most widely studied Mg-based alloys comprise AZ (Mg-Al-Zn system) and WE alloys (Mg-RE-Zr system) [85]. AZ alloys such as AZ31 (Mg-3Al-1Zn) and AZ91 (Mg9Al-1Zn) have excellent mechanical properties, but the high degradation rate and local toxicity of aluminum limit their clinical use [86]. On the other hand, WE43 alloy (Mg-4Y-3RE) is coated with a RE-oxide layer, improving corrosion resistance and biocompatibility [87]. WE43 alloy (Mg-3.5% Y-2.3% Nd-0.5% Zr, wt.%), MgYREZr alloy and Mg-Nd-Zn-Zr alloy were assessed in in vitro and in in vivo studies for bone repair, which resulted in good bone repair when used as pins or screws for bone fixation in orthopedic patients [88,89]. ZX00 (Mg-Zn-Ca alloy) is another resorbable alloy that revealed good results in pre-clinical studies on bone regeneration [90].

Recent clinical trials investigating the use of Mg and its alloys for stabilization of orthopedic fractures revealed excellent results in fracture reduction of displaced femoral neck fractures, hallux valgus and medial malleolar fractures, with the bone regeneration rates comparable to Ti screws [91,92,93,94,95]. Most of these trials investigated the use of MgYREZr alloy screws to stabilize unstable fractures. Due to the release of H_2_ ions due to corrosion, a radiolucent zone around screws was observed in the majority of postoperative radiological exams. However, no severe complications were observed [96].

Reports on the use of Mg and its alloys in the treatment of facial bone fractures are scarce. Traumatic injuries to the facial bones are among the most common injuries to the body, mostly reported in traffic accidents and interpersonal violence. Fractures in the maxillofacial area have a significant impact on patients’ appearance, speech and mastication [1,2]. The treatment of facial bone fractures requires the repositioning of fractured bone fragments to the anatomical state and osteosynthesis with Ti plates and screws. Ti plates and screws are used due to the excellent biocompatibility and biomechanical properties of Ti and usually are left for life [11]. However, they sometimes need to be extracted due to an infection or discomfort [66]. Biodegradable plates and screws may be beneficial in avoiding second-stage surgery. Biodegradable materials for use in the maxillofacial area must overcome some factors specific to this region. These include factors such as significant masticatory muscle forces, presence of saliva and intraoral pathogens. This is because most of the surgical interventions are performed through an intraoral approach, with different elastic modules of facial bones as well as various shapes of bones [80,97]. Biodegradable polymer fixation plates made from PLLA and PLGA have poor mechanical properties and may cause an inflammatory reaction [98]. Mg-based materials possess good mechanical strength and biocompatibility with proven clinical applications. However, the compressive yield strength of Mg-based alloys is lower than Ti alloys which questions their use for load-bearing fractures such as mandible fractures [99]. Pre-clinical studies revealed Mg-based materials as promising candidates for maxillofacial bone osteosynthesis [99,100,101,102,103,104,105,106,107,108,109,110] (Table 1).

Mandibular fractures are the most common fractures in the maxillofacial area, and their treatment consists of thick Ti plates and locking screws to restore the bone’s anatomical shape along with occlusion, and avoid postoperative movement of the fragments by heavy masticatory forces [102]. Only one animal study by Nujokat et al. used MgYZrRee (WE43) custom-made fixation plates and screws for the stabilization of mandibular osteotomy at the mandibular angle [103]. The results of this in vivo study proved good mechanical stability at the osteotomy site. However, the performed osteotomy was monocortical and did not represent a full bicortical fracture line. Mg screws were investigated in several studies and reported better mechanical properties compared to the polymeric material but lower mechanical and torsional strength than Ti controls [98,99,100]. Interesting are the results of Mg screws for stabilization of osteotomy lines for bilateral ramus sagittal split osteotomy (BSSO) performed for orthognathic surgery procedures where the mandibular setback or advance is performed to correct maxillofacial deformities. The results of two studies based on finite element modeling found the use of Mg or Mg-Ca-Zn screws could stabilize the osteotomy lines even with masticatory loading [100,101]. Further pre-clinical and clinical trials are needed in order to obtain an Mg-based fixation system for mandible fractures and osteotomies to overcome current disadvantages regarding mechanical stress and low torsional strength.

On the other hand, the results of animal studies on the fixation of the midface complex fractures are more promising. Midface fractures, mainly fractures of the maxilla and zygomatic bone, are load-shearing types of fractures where no significant masticatory forces are implied to reduce the stability of the fracture line. The use of WE43 plates and screws for the fixation of fractures in the midface resulted in good osteotomy lines stability, biocompatibility, and osseointegration [103,104,105,106]. The gas formation was observed for 12 weeks postoperatively without side effects on bone regeneration and wound healing, proposing that the material’s degradation rate is adequate. The use of PLLA-coated ZK60 plates and screws for fixation of Le Fort I osteotomy in beagles resulted in significant gas formation and local inflammation due to the fast biodegradation of the material [107]. Although ZK60 plates showed good mechanical properties, it seemed that PLLA coating failed to prevent the rapid absorption of the alloy due to micro-cracks on the surface [107]. Further research is needed to obtain alloys with more predictable rates of biodegradation and mechanical properties for these types of fractures. Fixation systems based on Mg materials used in these studies were thicker and had a bigger volume compared to Ti fixation systems, although there was no significant discomfort to the subjects

Promising results of pre-clinical studies have been published regarding the use of WE43 plates and screws for the fixation of fractures in the frontal bone [108,109]. The stability of the plates and biocompatibility were comparable to the Ti fixation system.

The repair of orbital fractures represents a significant challenge to the surgeons due to the proximity of intracranial structures, paranasal sinuses, the poor blood supply of the bones and osteoprogenitor cell insufficiency [110]. The thin bony walls of the orbit, especially the inferior and medial walls, are the most prominent locations for fractures. Blow-out fractures of the orbital floor are the most common fracture of the orbit. Current materials used for fracture reduction and reconstruction of the orbital volume are bioinert Ti meshes, plates, and polyethylene meshes. Zhang et al. developed Ca-P coated Mg-Zn-Gd scaffold to reconstruct a large defect of the medial orbital wall in a canine model [110]. The results showed excellent osteoconductivity, angiogenesis and bone regeneration with the scaffold. The authors observed no gas formation and orbital emphysema.

Only two clinical studies by Leonhardt et al. reported the effectiveness of Mg-based materials for the treatment of fractures in maxillofacial surgery [111,112]. These studies reported repositioning and fixation of mandibular condyle fracture with Magnezix^®^ CS 2.7 mm screw (MgYREZr alloy). The authors reported excellent stabilization of fragments and complete restoration of temporomandibular joint (TMJ) function. Gas formation around screws was reported and seen as radiolucent areas on control CBCT exams. One year follow-up was uneventful, and there was no need for screw removal (Table 2).

## 6. Mg-Based Materials for Soft Tissue Regeneration

The application of Mg and Mg-based materials for bone tissue regeneration is well-known. Several studies revealed Mg has a positive impact on the regeneration of soft tissue in the maxillofacial region.

Mg scaffolds induce cell proliferation, migration, and osteogenic differentiation of human dental pulp cells and participate in the process of pulp repair [113,114,115].

Mg ions have positive effects on the migration and adhesion of human fibroblasts and oral mucosa regeneration [116,117]. The effects of Mg on fibroblast activity could have a promising effect on the alteration of the Ti implant surface and promote soft tissue healing around the neck of the implant [118,119]. In an in vitro study by Okawachi et al. [120], hydrothermal treatment of Ti with an Mg solution improved the integration of gingival epithelial cells and fibroblasts with the Ti surface. Furthermore, Mg has antibacterial properties against common periodontal pathogens [78].

Previous studies reported biomimetic scaffolds with Mg nanoparticles combined with polymers had promising results for cartilage regeneration [86]. This scaffold may positively impact the treatment of TMJ disorders. Having in mind the joint cartilage is mainly fibrous, lacks blood supply, and has limited self-repair, as well as that the properties of Mg include anti-inflammatory effect, enhanced synthesis of the cartilage matrix, promotion of chondrocyte proliferation, and enhanced chondrogenic differentiation of hBMSCs, the application of Mg-based materials may be a promising new strategy in the treatment of chronic TMJ conditions [121,122,123].

It is known that Mg ions are involved in neurotransmission through the n-methyl-D-aspartic acid receptor and the inhibition of the production of glutamatergic excitation signals [124]. Several pre-clinical studies showed that Mg supplementation positively affected sciatic nerve regeneration and repair [125,126]. One animal study revealed that oral or intravenous Mg might reduce the signs of trigeminal neuralgia [127]. Sensory nerve neuropathies in the maxillofacial area may cause significant impairment to patients’ quality of life. Primary trigeminal neuralgia is a form of chronic neuropathic pain that affects branches of the trigeminal nerve. Current treatment procedures involve therapeutic drugs, and surgical interventions when drug treatment is ineffective. Peripheral nerve branches in the maxillofacial area may be injured during surgical interventions: great auricular nerve during parotidectomies, inferior alveolar nerve during operation of cysts or tumors in the lower jaw, and infraorbital nerve during surgical procedures in the maxilla. Mg supplementation may be beneficial in the treatment of these neuropathic conditions [124].

In addition, the trauma to the peripheral motoric branches of the facial nerve during parotid gland surgery or mastoidectomy can result in facial paralysis. Restoring the function of motor nerves is much more difficult and uncertain compared to sensory nerves [128]. Gougoulias et al. reported in an in vivo study that subcutaneous injection of Mg in neonatal rats reduced motor neuron death after sciatic nerve axotomy [129]. In vitro studies showed Mg ions could promote the proliferation of neural stem cells [130]. Further studies are needed to evaluate the role of Mg in sensory and motor nerve repair.

Table 3 summarizes application areas of Mg-based materials in reconstructive oral and maxillofacial surgery.

## 7. Conclusions

Mg-based materials have been extensively studied for their use in biomedicine in the past decade. Mg-based materials represent a very promising group of biomaterials for application in reconstructive medicine. Mg has an essential role in cell metabolism, and it is involved in more than 300 enzymatic processes. Mg-based materials are biodegradable, biocompatible, with elastic modulus similar to that of bone and with a positive effect on bone regeneration. In the field of reconstructive oral and maxillofacial surgery, its positive effects were reported in the areas of guided bone regeneration, improvement of dental implant osseointegration, fixation of facial bone fractures and regeneration of soft tissues. Due to the positive effect on bone repair and differentiation of osteoblasts, Mg-based materials were successfully evaluated in clinical studies for guided regeneration of jaw bones. In vitro and in vivo studies reported improved osseointegration when Mg coating was applied to the Ti implant surface. Clinical studies on the application of Mg-based materials for the treatment of maxillofacial fractures have been published, and further research is needed to develop the Mg alloy with adequate mechanical strength and degradation rate. Further research is still needed to improve the characteristics of Mg-based materials for application in the maxillofacial area.

## Figures and Tables

**Table 1:** Magnesium (Mg)-based materials for osteosynthesis of maxillofacial bones.

Reference	Study	Materials	Fixation Type	Methodology	Evaluation	Results
Lee et al. [100]	Finite element modeling	Mg (pure) Polymer Ti	Screw	Bilateral mandibular ramus sagittal split osteotomy	Stress distribution	Mg screws maintained stability at osteotomy sites superior to the polymer material
Lee et al. [101]	Finite element modeling	Mg-Ca-Zn alloy Polymer Ti	Screw	Bilateral mandibular ramus sagittal split osteotomy	Stress distribution	Mg-Ca-Zn screws maintained stability at osteotomy sites and displayed masticatory loading superior to the polymer material
Schaller et al. [102]	Animal experiment (minipigs)	WE43 alloy	Rivet	Implantation on mandibular angle	Histology Micro-CT	Sufficient stability of the rivets during 12–24 weeks
Naujokat et al. [103]	Animal experiment (minipigs)	WE43 alloy	Plate + screws	Unicortical osteotomy at mandibular angle	Histology Micro-CT	Sufficient stability of the plates and screws for 8 weeks, no side effects
Henderson et al. [99]	Animal experiment (rabbits)	Mg AZ31 alloy Stainless steel	Screw	Implantation on mandibular angle	Histology Micro-CT	Sufficient stability of the screws, physiological bone remodeling
Byun et al. [104]	Animal experiment (beagles)	WE43 Ti	Plate + screws	Le Fort I osteotomy of the maxilla	Histology Micro-CT	Sufficient stability of the plates and screws for 24 weeks; significant gas formation in the first 12 weeks
Byun et al. [105]	Animal experiment (beagles)	ZK60 coated with PLLA	Plate + screws	Le Fort I osteotomy of the maxilla	Micro-CT	Rapid biodegradation of ZK60 resulted in insufficient results
Schaller et al. [106]	Animal experiment (minipigs)	WE43 Polymer (PLGA)	Plate + screws	Osteotomy at supraorbital rim and zygomatic arch	Histology Micro-CT	Sufficient stability of the plates and screws in the midface region
Kim et al. [107]	Animal experiment (beagles)	WE43 polymer	Plate + screws	Osteotomy at zygomatic arch	Histology Micro-CT	Sufficient stability, biocompatibility and osteogenic activity of the plates and screws in the midface region
Naujokat et al. [108]	Animal experiment (minipigs)	WE43 Ti	Plate + screws	Frontal bone osteotomy	Histology Micro-CT	WE43 sufficient stability of the plates and screws in the calvaria compared to Ti
Schaller et al. [109]	Animal experiment (minipigs)	WE43 Ti	Plate + screws	Frontal bone osteotomy	Histology Micro-CT	WE43 sufficient stability of the plates and screws in the calvaria compared to Ti
Zhang et al. [110]	Animal experiment (canines)	Ca-P coated Mg-Zn-Gd scaffold Ti	Mesh	Defect of the medial orbital wall	Histology Micro-CT	Ca-P coated Mg-Zn-Gd scaffold resulted in excellent bone regeneration, no gas formation

Mg—Magnesium; Ti—Titanium; Ca—Calcium; Zn—Zinc; Micro-CT—Micro-computed tomography; PLLA—Poly(L-lactic acid); PLGA—Poly(lactic-*co*-glycolic acid); Ca-P—Calcium phosphate; Gd—Gadolinium.

**Table 2:** Clinical studies on magnesium (Mg)-based material for stabilization of fracture of the mandibular condyle.

Reference	Study	Fracture Pattern	N	Material	Results	Complications
Leonhardt et al. [111]	Case series	Displaced fractures of the condylar head with a loss of height on the mandibular ramus, and clinical signs such as pain, malocclusion, and jaw movement, limited excursions	4 patients with unilateral fractures 1 patient with bilateral fracture	Magnezix^®^ CS 2.7 mm screw (Syntellix AG, Hanover, Germany)	Stabilization of fracture, restored function of TMJ, no gass formation during 3 months	One accidental fracture of the screw which was replaced
Leonhardt et al. [112]	Retrospective observational study	Displaced fractures of the condylar head with a loss of height on the mandibular ramus, and clinical signs such as pain, malocclusion, and jaw movement, limited excursions	6 patients	Magnezix^®^ CS 2.7 mm screw (Syntellix AG, Hanover, Germany)	Restoration of occlusion and function of TMJ, gas lacunas visible for 6 months afterwards filled with bone, partial resorption of screws in first year	none

TMJ—Temporomandibular joint.

**Table 3:** Overview of applications of magnesium (Mg)-based materials in reconstructive oral and maxillofacial surgery.

Application		Study			Advantages	Disadvantages	Future Directions
		In vitro	In vivo	Clinical			
Fracture reduction	Mandible fracture	+	+	+	-biocompatibility -degradation -elastic modulus -mechanical properties -no second stage surgery	-low resistance to masticatory stress	-improvement of mechanical resistance for load-bearing fractures -development of Mg alloys with predictive degradation rate
	Midface fracture	+	+	−		-uncontrolled degradation rate	
	Frontal bone fracture	+	+	−		-uncontrolled degradation rate	
GBR	Scaffolds	+	+	+	-biocompatibility -osteoconductivity -bone repair	-low porosity	-improvement of 3D porosity
	Membrane	+	+	−	-biocompatibility -degradation-mechanical properties -osteogenic effect -small and large bone defects -antibacterial activity	-uncontrolled degradation rate	-improvement of mechanical properties and degradation rate
Oral implantology		+	+	−	-biocompatibility -degradation-osteoblastic differentiation -antibacterial activity	-degradation rate	-need for clinical trials -development of techniques for Mg coating
Soft tissue regeneration	TMJ	+	−	−	-protective effect on cartilage	-no data on TMJ regeneration	-no trials on the possible use on TMJ cartilage regeneration
	Dental pulp	+	−	−	-dental pulp repair		-no trials on the preclinical or clinical use
	Oral mucosa	+	−	−	-fibroblast activation -mucosa regeneration -antibacterial properties		-possible use in dental implantology
	Nerve tissue	+	+	−	-nerve regeneration		-possible use in sensitive nerve neuropathy

GBR—Guided bone regeneration; TMJ—Temporomandibular joint.

## Data Availability

Not applicable.
